# Ultrasound combined with Adenosine 5′-Monophosphate Treatment: A Strategic Approach for enhancing the tenderness of chicken wooden breast meat

**DOI:** 10.1016/j.ultsonch.2025.107284

**Published:** 2025-02-18

**Authors:** Xiang Yu, Yanli Feng, Wenhan Ma, Xue Xiao, Jun Liu, Weiwei Dong, Yuanliang Hu, Huan Liu

**Affiliations:** a Hubei Key Laboratory of Edible Wild Plants Conservation & Utilization, College of Life Sciences, Hubei Normal University, No. 11, Cihu Road, Huangshi City 435002 Hubei Province, China; b Hubei Engineering Research Center of Special Wild Vegetables Breeding and Comprehensive Utilization Technology, Hubei Normal University, Huangshi 435002, China

**Keywords:** Chicken wooden breast, Ultrasound, Adenosine 5′-monophosphate, Tenderness

## Abstract

This study aimed to evaluate the effects of ultrasound and adenosine 5′-monophosphate (AMP) treatments on the quality characteristics and tenderness of chicken wooden breast (CWB). Compared to normal breast, CWB exhibits distinct quality characteristics, including increased weight, higher pH, pale color, and a firmer texture. It was found that ultrasound, AMP, and their combined application significantly reduced the shear force of CWB (*p* < 0.05), effectively improving its tenderness. The combined treatment of ultrasound and AMP significantly decreased the filtering residues of myofibrillar proteins (MPs) and increased myofibrillar fragmentation index (*p* < 0.05). MPs structure analysis showed that the combined ultrasound and AMP treatment facilitated the degradation of tropomyosin, the transformation of α-helix into β-sheet, and decreased intensity of tryptophan fluorescence, promoting MPs degradation and improving CWB tenderness. Pathological analysis and scanning electron microscopy also observed muscle fiber damage and the loss of myofibrillar membrane integrity following the combined treatment. These findings highlight the potential of AMP and ultrasound treatments in the tenderization process of CWB.

## 1 Introduction

Chicken, owing to its lack of regional, dietary, ethnic, and cultural limitations, serves as the foremost source of protein for the global population [1]. However, a type of abnormal-quality chicken breast is prevalent worldwide, characterized by the enlargement, pallor, and hardening of the pectoralis major, with or without white striations, as well as highly elastic breast muscles resembling rubber in appearance [2], [3]. This meat, defined as chicken wooden breast (CWB), not only negatively impacts the visual characteristics of the pectoralis major, reducing consumer acceptance and purchase intent, but also decreases product yield and edible quality during processing, leading to economic losses [3], [4]. CWB is characterized by substandard quality traits, including poor color, water-holding capacity and texture. Despite numerous attempts by researchers to enhance the quality of CWB, the outcomes have been largely unsatisfactory. For instance, the incorporation of inorganic salts has proven ineffective in improving water-holding capacity [5], and heat-induced treatments failed to enhance its gelation properties [6]. In general, the processing characteristics of CWB are inferior, posing significant challenges for poultry processing industries. Consequently, there is an urgent need for pragmatic and effective strategies to modify the processing properties of CWB.

CWB, refers to the fibrous hardening of chicken breast tissue, which plays a crucial role in affecting tenderness. Tenderness, due to the complex regulatory mechanisms and modification behaviors involved in its formation, becomes a core attribute of CWB quality. For instance, CWB as “white meat,” does not exhibit significant color differences after cooking or processing, its abnormal flavor can be masked or enhanced through the use of spices [7]. Notably, tenderness, which is a sensitive attribute during mastication, fracture, or cutting, is a complex sensory experience perceived in the mouth. Due to the difficulty in modifying tenderness, which plays a pivotal role in influencing consumer preferences for meat products, it is particularly significant for processed products. Currently, studies have reported the use of CWB mixed with normal chicken meat in product manufacturing. When the proportion of CWB exceeded 20 %, sausages exhibited a decline in quality [8]. Although patties containing 50 % CWB showed a 70 % acceptance rate, this was still lower than that of the normal chicken meat [9]. Given the processing characteristics of CWB, its applications are highly limited. Therefore, there is an urgent need to identify novel methods to enhance its tenderness and improve its sensory attributes.

The tenderization process is intricate, involving numerous interrelated mechanisms, including the degradation of collagen, the separation of muscle fiber bundles, the rupture of sarcomeres, and the alterations in protease activity and energy metabolism that occur during aging [10]. Ultimately, the essence of this process lies in the interactions between myofibrillar proteins (MPs) and the ultrastructure of the muscle tissue [11]. Endogenous enzymes and apoptosis-induced degradation of MPs are generally considered the most effective pathways for improving meat tenderness [10]. Adenosine 5′-monophosphate (AMP) holds significant potential for enhancing muscle quality. AMP activates the activity of AMP kinase (AMPK) and caspase-3 by binding to the specific γ subunit of AMPK, thereby upregulating apoptosis and muscle protein degradation. Simultaneously, AMP enhances the dissociation of actomyosin, the disruption of myofibrils, and the release of actin from MPs, all of which contribute to postmortem tenderization. It is important to note that AMP, through deamination, is converted into inosine monophosphate (IMP), which enriches the umami and flavor of meat. Furthermore, exploring novel technologies to accelerate quality transfer and improve meat tenderization is imperative [12]. Ultrasound, as an innovative food processing technology, offers numerous advantages, such as high efficiency, safety, deep penetration, and low energy consumption. Ultrasound induced cavitation, resulting in muscle fiber rupture and protein denaturation, and enhanced water migration, thus making it widely applied in meat processing [13]. Overall, the combined use of AMP and ultrasound technology holds considerable potential for improving the quality of CWB.

Although AMP and ultrasound technology have been applied in meat research, the effects of ultrasound combined with AMP treatment on the tenderness and quality of CWB have not been reported. This study aimed to investigate the changes in shear force, myofibrillar fragmentation index (MFI), MPs structure, and tissue morphology following ultrasound combined with AMP treatment of CWB. By delving into the mechanisms underlying the tenderization of CWB, this research evaluated the potential of ultrasound and AMP in improving CWB quality. The findings may offer new insights into enhancing muscle quality and developing effective tenderization methods.

## 2 Materials and methods

### 2.1 Material preparation

Chicken wooden breast (CWB) was purchased from Cargill Protein and Nutrition Technology Co., Ltd. (Chuzhou, China) and sourced from 45-day-old white-feather broilers. The selection and identification of CWB were carried out using visual inspection and pressing tests, following the methods reported by Wang et al [14].

### 2.2 Experimental design and tenderization treatment

The tenderization of CWB was performed using a liquid curing method. The CWB was placed in a polypropylene container containing the curing solution (5 % NaCl). The chicken breast was immersed in the curing solution at a ratio of 1:1.5 (***w/v***) for 30 min. The tenderization treatments were divided into five groups: (***i***) control group (CK) with no liquid curing; (***ii***) curing solution treatment (W); (***iii***) 300 W ultrasound treatment for 5 min (W + U); (***iv***) curing solution with 32 mM Adenosine 5′-monophosphate (AMP) (A); and (***v***) curing solution with 32 mM AMP, followed by 300 W ultrasound treatment for 5 min (A + U) [15].

### 2.3 Meat quality measurements

#### 2.3.1 pH and whiteness

The pH value of CWB was measured using a portable puncture type pH meter (Testo 205, Testo SE & Co., Baden-Württemberg, Deutschland). Following the manufacturer’s instructions, the probe of the pH meter was inserted into the chicken breast, and the readings were recorded. The surface color of the chicken breast was measured using a colorimeter (CR-10, Konica Minolta, Tokyo, Japan) to obtain the whiteness (L* value).

#### 2.3.2 Compression force

The compression force of the chicken breast was measured using a texture analyzer (FTC Corporation, Washington, USA) with a 12 mm cylindrical probe applied to the head of the chicken breast. The test parameters were set as follows: pre-test downward speed of 1.0 mm/s, testing speed of 1.0 mm/s, post-test return speed of 1.0 mm/s, a sample compression strain of 30 %, and a trigger force of 0.2 N.

#### 2.3.3 Shear force

The shear force of the chicken breast was measured using a texture meter (FTC Co., Washington, USA), following the method of Cai et al. [16]. Specifically, the steamed chicken breast was sliced into 3 × 1 × 2 cm pieces along the muscle fiber direction, and the maximum shear force perpendicular to the fibers was recorded. A 250 N force was applied using a mass spectrometer fitted with a 500 N pressure transducer, with an initial force of 0.2 N. The test and post-test speeds were set to 2 mm/s, and the return distance was 50 mm.

### 2.4 Structural analysis of myofibrillar proteins (MPs)

#### 2.4.1 Preparation of MPs

Chicken breast MPs were extracted following the method of Guo et al [17], with slight modifications. The chicken breast was minced using a meat grinder, and 5 g of meat paste was added to four volumes (*w/v*) of 20 mM phosphate-buffered saline (PBS, containing 0.1 M NaCl, 2 mM MgCl _2_ and 1 mM EDTA, pH 7.0) for homogenization (Denson Biotechnology Co., Ltd., Shanghai, China). The mixture was homogenized twice for 12 s each, followed by centrifugation at 2000 × g, 4 °C for 15 min. After each homogenization, the supernatant was discarded to obtain the precipitate. The precipitate was then resuspended in four volumes of 0.1 M NaCl, and the homogenization and centrifugation steps were repeated twice. The final homogenate was filtered through four layers of 80-mesh gauze to remove connective tissue, and the precipitate obtained after centrifugation constituted the MPs extract.

#### 2.4.2 Filtering residues

Chicken breast MPs were extracted according to the procedure of Guo et al. [17], with slight modifications. The breast meat was minced and mixed with four volumes (*w/v*) of 20 mM PBS (0.1 M NaCl, 2 mM MgCl _2_, and 1 mM EDTA, pH 7.0). The mixture was homogenized twice for 12 s each using a meat grinder (Denson Biotechnology Co., Ltd., Shanghai, China), then centrifuged at 2000 × g for 15 min at 4 °C. Following each homogenization, the supernatant was discarded, and the precipitate was resuspended in four volumes of 0.1 M NaCl. The homogenization and centrifugation were repeated twice more. Finally, the homogenate was filtered through four layers of 80-mesh gauze to remove connective tissue, and the MPs extract was collected from the resultant precipitate.

#### 2.4.3 Myofibrillar fragmentation index (MFI)

The MPs extract was diluted to a concentration of 0.5 mg/mL using PBS, and absorbance was measured at 540 nm. The MFI was calculated as 200 times the recorded absorbance value [19].

#### 2.4.4 Sodium dodecyl sulfate–polyacrylamide gel electrophoresis (SDS-PAGE)

The procedure outlined by Zhang et al. was adapted with minor modifications [20]. Briefly, the MPs concentration was adjusted to 5 mg/mL in PBS, and the protein samples were combined with loading buffer in a 1:1 (*v/v*) ratio. The mixture was then heated at 100 °C for 5 min. A molecular weight marker (10 kDa to 150 kDa) was used as a standard. SDS-PAGE was performed with a 5 % stacking gel and a 12 % separating gel. The stacking gel was run at a constant 80 V, while the resolving gel was operated at 150 V. After electrophoresis, the gels were stained with Coomassie Brilliant Blue R-250 on a shaker for 2 h, then destained until clear protein bands were visible. Gel images were captured using a gel imaging system (Bio-Rad, CA, USA), and band intensities were quantified using ImageJ software to obtain grayscale values for analysis.

#### 2.4.5 Circular dichroism (CD)

The CD was analysed based on the experimental procedures of Liu et al [21], with slight modifications. MPs was diluted to a concentration of 0.2 mg/mL using 20 mM PBS, pH 7.0 and transferred into a quartz CD cuvette with a 1.0 mm optical path length. The PBS was used as the spectral baseline. The scanning speed and bandwidth of the CD (Jasco 810, Jasco Corp., Tokyo, Japan) spectrometer were set to 100 nm/min and 2.0 nm, respectively. The percentages of secondary protein structures (α-helix and β-sheet) were calculated using CD Pro software.

#### 2.4.6 Fluorescence spectra

Fluorescence spectra of MPs were measured according to the method of Zhang et al. [22], with slight adjustments. The MPs solution was diluted to 0.5 mg/mL in PBS, and spectra were acquired in the 300–400 nm range using a fluorescence spectrophotometer (Hitachi, Tokyo, Japan). The excitation was set to 283 nm, with both excitation and emission slit widths at 10 nm.

### 2.5 Carbonyl and sulfhydryl content

The levels of carbonyl and sulfhydryl groups in MPs were quantified following the protocols provided by the kit manufacturer (Jiancheng BRI., Jiangsu, China). The assay procedures were executed meticulously to ensure accuracy, with strict adherence to the manufacturer's guidelines regarding reagent preparation, incubation times, and measurement parameters.

### 2.6 Determination of surface hydrophobicity

The MPs were prepared at a concentration of 5 mg/mL in PBS. Subsequently, 1 mL of the MPs solution (with PBS serving as the control) was combined with 200 μL of bromophenol blue (BPB) solution at 1 mg/mL and thoroughly mixed. The mixture was then subjected to shaking for 10 min in the dark, followed by centrifugation at 4000 g, 4 °C for 15 min. After centrifugation, the supernatant was diluted tenfold with PBS, and its absorbance was recorded at 595 nm [23]. The surface hydrophobicity (SH) of the MPs was determined using the following formula:

$$\text{SH (}\mu \text{g) = 200}\times \left({Absorbance}_{MPs}-{Absorbance}_{PBS}\right)/{Absorbance}_{PBS}$$

### 2.7 Disulfide bond content

The disulfide bond content was measured using the method of Ma et al [24], with some modification. Briefly, 100 μL of MPs extract was mixed with 3 mL of NTSB solution (containing 1 M Na_2_SO_3_, 10 mg/mL DNTB, pH 7.25) and incubated in a 38 °C water bath for 1 h. Afterward, 1:100 (***v/v***) of the reaction solution (comprising 2 M guanidine thiocyanate, 50 mM glycine, 100 mM Na_2_SO_3_, 3 mM EDTA, pH 9.5) was added, followed by vortex mixing. The mixture was incubated at room temperature in the dark for 25 min, and absorbance at 412 nm was measured. PBS (20 mM, containing 0.1 M NaCl, 2 mM MgCl_2_, 1.0 mM EDTA, pH 7.0) served as the blank control. The disulfide bond content was calculated using the following formula:

$$C_{\mathrm{Disulfidebond}}\left(\frac{\mathrm{nmol}}{\mathrm{mg}}\right)=\left(A_{412}\times 10^{6}\right)/\left(13900\times C\right)\times Dilutionfactor$$

where A_412_ is the absorbance at 412 nm, 13,900 L/(mol·cm) is the molar extinction coefficient, and C is the protein concentration of the MPs.

### 2.8 Hematoxylin eosin (HE) staining

Meat samples (1 cm × 1 cm × 1 cm) were immersed in 10 times their volume of 4 % paraformaldehyde fixative for 24 h. After fixation, the samples were paraffin-embedded, sectioned, and stained with hematoxylin and eosin (HE). Following dehydration in ethanol and xylene, the sections were mounted with neutral gum. Tissue microstructure was examined under a microscope (Nikon, Tokyo, Japan) at 200 × magnification [25].

### 2.9 Scanning electron microscopy (SEM)

SEM measurements were performed with slight modifications based on the method outlined by Cai et al [16]. Pre-processing steps, including sample fixation, ethanol dehydration, cleaning, freeze-drying, sectioning and gold plating. Microstructure images of the samples were obtained by scanning electron microscopy (TM3030, Hitachi, Japan) at 500 × magnification.

### 2.10 Statistical analysis

Results are presented as mean ± standard deviation. Graphs were generated using Origin 2018b, and statistical analyses were carried out with SPSS 24.0 (IBM Corp., Armonk, NY, USA). Significance was determined by one-way ANOVA, followed by Duncan's multiple range test for pairwise comparisons (Origin Lab Corp., Northampton, MA, USA), with *p* < 0.05 considered statistically significant.

## 3 Results and discussion

### 3.1 Quality characteristics

The quality indicators of chicken breast samples from normal chicken breast (NB) and chicken wooden breast (CWB), were systematically analyzed. The mean weight of the CWB samples (353.10 ± 9.81 g) was significantly higher than that of the NB samples (240.19 ± 7.77 g, *p* < 0.05). Similarly, the pH value of CWB samples (6.14 ± 0.11) was higher than that of NB sample (5.62 ± 0.05, *p* < 0.05). CWB samples exhibited a higher L* value (56.73 ± 1.18) than NB samples (53.23 ± 1.09, *p* < 0.05), indicating changes in surface color. Furthermore, the compression force, reflecting tissue hardness, was markedly elevated in CWB samples (16.57 ± 1.99 N) compared to NB samples (8.37 ± 0.30 N, *p* < 0.05), suggesting significant textural differences between the two treatments. These findings highlight the pronounced differences in weight, pH, color, and textural properties between NB and CWB. Specifically, these results demonstrated that CWB was associated with poorer meat quality, including reduced weight, pale color, and a tougher, more difficult-to-chew texture.

**Fig. 1:**
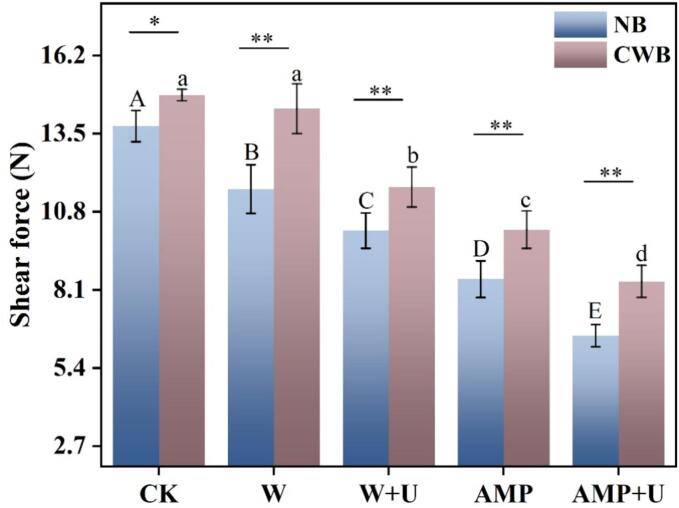
The impact of AMP and ultrasound treatments on the shear force of chicken breast. Significant differences within groups are denoted by *p* < 0.05, where “*” indicates *p* < 0.05 and “**” indicates *p* < 0.01.

As shown in Table 1, CWB exhibited markedly inferior apparent characteristics, thereby prompting further exploration of the potential to enhance its tenderness traits through the application of adenosine monophosphate (AMP) and ultrasound treatment. Shear force is an evaluative measure of muscle tenderness, and as shown in Fig. 1, the pressure loss of CWB was significantly higher than that of NB (*p* < 0.05). An intriguing phenomenon was observed: with the exception of soaking in 5.5 % sodium chloride solution, all treatments significantly improved the shear force of chicken breast (*p* < 0.05). For CWB, the effect of the single AMP treatment outperformed that of ultrasound treatment, with shear force reductions of 4.65 and 3.18, respectively. The combination of ultrasound and AMP treatment resulted in the most pronounced decrease in pressure loss, with values of 6.52 in NB and 8.40 in CWB (*p* < 0.05). These results underscore the efficacy of AMP and ultrasound treatments, particularly when combined, in mitigating pressure loss in CWB tissues.

**Table 1:** Differences in quality indicators between normal (NB) and woody (CWB) chicken breasts.

Quality indicators	Chicken breast category	
	Normal chicken breast (NB)	Chicken wooden breast (CWB)
Weight (g)	240.19 ± 7.77^b^	353.10 ± 9.81^a^
pH	5.62 ± 0.05^b^	6.14 ± 0.11^a^
L*	53.23 ± 1.09^b^	56.73 ± 1.18^a^
Compression force (N)	8.37 ± 0.30^b^	16.57 ± 1.99^a^

**Note:** Different lower case letters in the same row represent significant differences (*p* < 0.05).

### 3.2 Degree of muscle fibre destruction

**Fig. 2:**
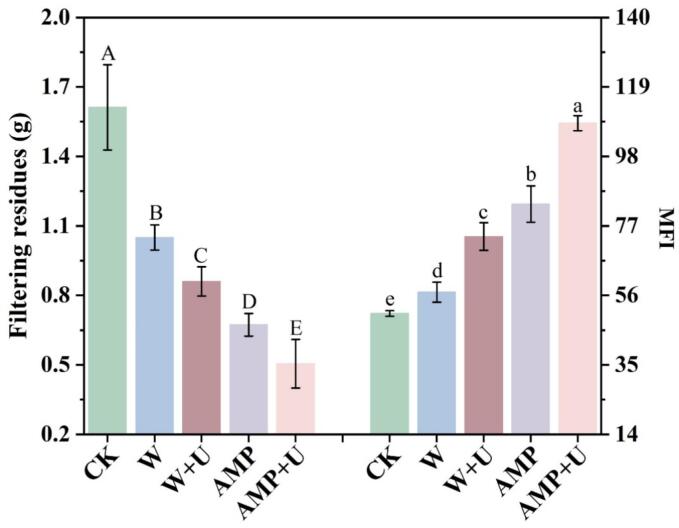
Effect of AMP and sonication on CWB filtering residues. Significant differences within groups are denoted by *p* < 0.05.

The filtering residues consists of a mixture of myofibrillar proteins (MPs) and sarcolemma, commonly used to evaluate the level of insoluble proteins [18], [25]. In the case of woody breast, the lignification of muscle fibers reduces protein solubility, and the filtered residue can also serve as an indicator of the degree of protein lignification. As shown in Fig. 2, the control exhibited the highest value for filtration residue (1.61 ± 0.18 g), and the lowest MFI (50.56 ± 3.66). Ultrasonic treatment, whether applied individually or in combination, significantly reduced the amount of filtered residue while markedly increasing the Myofibrillar fragmentation index (MFI) (*p* < 0.05), suggesting a synergistic effect between the two interventions.

MFI represents the proportion of myofibrils with a length shorter than four sarcomeres, and its increase indicates that the integrity of the internal myofibrillar structure has been substantially disrupted [26], [27]. Barekat et al. proposed that ultrasound contributed to meat tenderization through two primary mechanisms [18]. Firstly, the mechanical effects of ultrasound disrupt the cell membrane, resulting in structural damage to MPs. Secondly, ultrasound facilitates the transfer of MPs substrates to proteolytic enzymes, thereby triggering proteolytic activation and enhancing enzymatic reactions. Consequently, as shown in Fig. 2, the combination of ultrasound and AMP demonstrated the most effective treatment. This superior outcome is attributed to AMP’s ability to further promote postmortem tenderization mechanisms through the upregulation of apoptosis and structural alterations in muscle proteins. Specifically, AMP facilitates apoptosis by activating AMPK and regulating downstream signaling molecules [28]. Moreover, AMP directly induces dissociation of actomyosin into myosin and actin, thereby enhancing the MFI through the actomyosin dissociation pathway [29]. It is noteworthy that an interesting phenomenon was observed from the trends in filtration residue and MFI changes, namely that AMP treatment induced a greater degree of myofibrillar fragmentation compared to ultrasound. As explained by Barido et al., AMP disrupts the phospho-chain linkage, ultimately altering the structure of muscle cells and the integrity of sarcomeric proteins, resulting in more efficient degradation [12]. Chen et al. also reported that AMP induced endogenous degradation of MPs, specifically targeting the I band and Z-line [30]. Therefore, it is hypothesized that this phenomenon arises because ultrasound damages the encapsulation of myofibers, whereas AMP improves the tenderness of CWB by promoting endogenous myofiber damage.

### 3.3 Structure of MPs

**Fig. 3:**
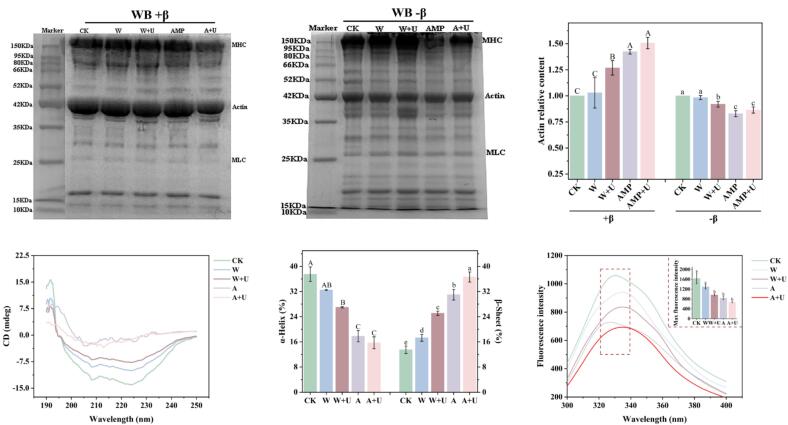
Effects of different treatments on the structure of myofibrillar proteins (MPs). (a) SDS-PAGE profile of MPs under reducing conditions. (b) SDS-PAGE profile of MPs under non-reducing conditions. (c) Relative content of actin. (d) Circular dichroism spectrum of MPs. (e) α-helix and β-sheet content of MPs. (f) Fluorescence spectrum of MPs. Significant differences within groups are denoted by *p* < 0.05.

SDS-PAGE analysis enables the identification of changes in the molecular weight of MPs through protein labeling, particularly the breakdown of tropomyosin into actin and myosin. Actin, a low molecular weight protein (approximately 43 kDa), can be easily identified by SDS-PAGE analysis, and thus the content of free actin is commonly used as an indicator of the dissociation of tropomyosin [31]. Meanwhile, Liu et al. reported that the dissociation of tropomyosin enhanced meat tenderness [32]. As shown in Fig. 3**a-c**, under both reducing and non-reducing conditions, the protein bands remained intact, with no band disruption observed after the treatments. This indicates that AMP (A) and ultrasound (U) treatments did not alter the subunit composition of the proteins, consistent with the findings of Cao et al [19]. Relative actin content changes (Fig. 3**c**) showed that after AMP and ultrasound treatments under reducing conditions, the content of actin significantly increased (*p* < 0.05). This suggested that both AMP and ultrasound treatments promoted the dissociation of tropomyosin into actin and myosin. This could be due to AMP specifically binding to large amounts of tropomyosin, facilitating its dissociation. Concurrently, ultrasound aids in the dissociation of tropomyosin by AMP, with the two exhibiting a synergistic effect. In summary, both AMP and ultrasound treatments improve the tenderness of CWB, with the combined treatment achieving a superior tenderization effect (Fig. 1, Fig. 2).

**Fig. 4:**
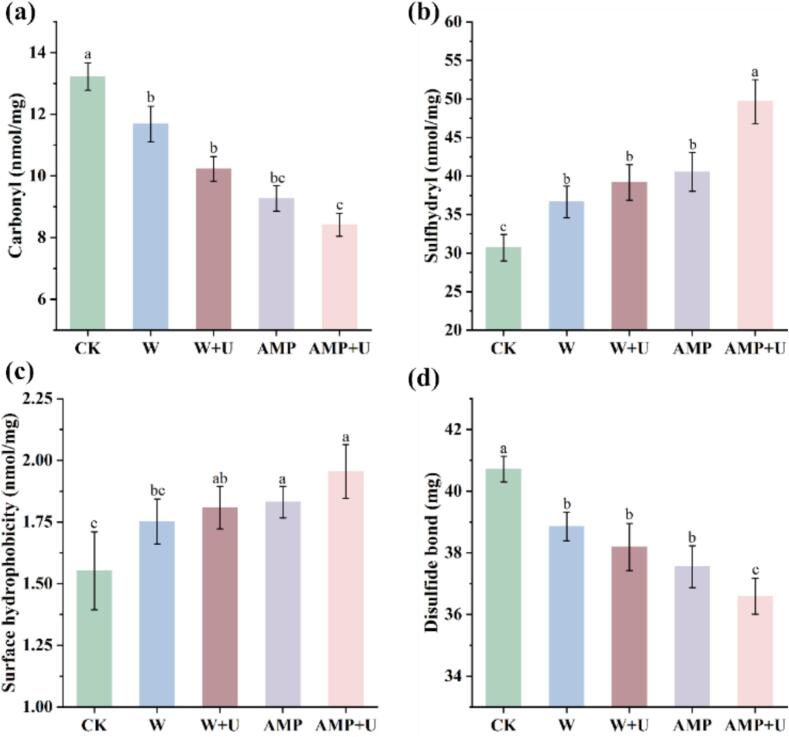
The impact of different tenderization methods on the intermolecular forces of myofibrillar proteins. (a) Effect on MPs carbonyl. (b) Effect on protein sulfhydryl. (c) MPs surface hydrophobic interactions. (c) Effect on MPs disulfide bonds. Significant differences within groups are denoted by *p* < 0.05.

As shown in Fig. 3**d**, the CD spectrum of MPs in the control group exhibited two prominent negative bands at 208 nm and 225 nm, indicating the dominance of an α-helical conformation due to the coiled-coil α-helical structure of the myosin tail [30]. Following ultrasound and AMP treatments, the characteristic helical patterns at 208 nm and 225 nm were notably diminished, suggesting a reduction in the α-helix secondary structure. Fig. 3**e** shows a significant decrease in α-helical content, decreased from 37.5 % to 15.7 %. Concurrently, an increase in the β-sheet content of MPs was observed, increased from 13.5 % to 36.6 % (Fig. 3**e**). Intrinsic tryptophan fluorescence can be used to characterize the tertiary structure of proteins, as it is particularly sensitive to the polarity of the microenvironment surrounding tryptophan residues. Generally, tryptophan residues are located in the protein core (hydrophobic environment), and folded, with high quantum yield, and exhibit characteristic fluorescence intensity [30]. As shown in Fig. 3**f**, the tryptophan fluorescence spectra of MPs exhibited a peak fluorescence intensity between 320–340 nm, and different treatments led to changes in the position and microenvironment of endogenous fluorescent chromophores within the amino acids. Compared to the control group, the tryptophan fluorescence intensity of MPs treated with ultrasound and AMP significantly decreased, particularly when ultrasound and AMP were combined. This may be because ultrasound and AMP treatments promote protein structural expansion and unfolding, causing tryptophan residues to be exposed and disrupting hydrophobic interactions, thus reducing fluorescence intensity. Simultaneously, the maximum fluorescence peak shifted noticeably to the right, suggesting that the combined treatment may expose hydrophobic groups in MPs [27]. Cai et al. reported that tryptophan oxidation and protein unfolding were also significant factors contributing to the reduction in fluorescence intensity [16]. This result was further supported by the results for protein carbonyl and thiol groups (Fig. 4 a and b).

### 3.4 Intermolecular interactions of MPs

The levels of carbonyl and sulfhydryl are commonly used as markers to characterize protein oxidation [33]. As shown in Fig. 4a and b, all treatments resulted in significant differences compared to the control group, specifically manifesting as a reduction in carbonyl levels and an increase in sulfhydryl levels (*p* < 0.05), the combined treatment of AMP and ultrasound notably reducing the carbonyl content from 13.22 to 8.42 nmol/mg, while simultaneously increased the sulfhydryl content from 30.69 to 49.68 nmol/mg. However, no significant differences were observed between the treatments with saline, ultrasound alone, and AMP alone (*p* > 0.05), suggesting that these individual treatments do not promote the oxidation of MPs. In contrast, the combined treatment significantly inhibited the oxidation of MPs (*p* < 0.05). Protein oxidation induces various alterations in proteins, including the formation of amino acid and protein polymers, a decrease in solubility, and an increase in carbonyl content. Protein radicals are formed when the side chains of proteins react with free radicals, which then interact with oxygen to generate peroxyl radicals that decompose to produce carbonyl derivatives [33]. The observed reduction in carbonyl levels (Fig. 4**a**) indicates the inhibition of protein oxidation. Additionally, no formation of protein polymers caused by oxidation was observed in the SDS-PAGE analysis (Fig. 3**)**. Reduction of sulfhydryl content is one of the key characteristics of protein alterations under oxidative conditions, with an increase in thiol levels indicating a reduction in oxidation. Sulfhydryl is highly susceptible to attack by reactive oxygen species (ROS), which can convert them into disulfide bonds, sulfinic acids, sulfonic acids, or nitrosylated species [34]. The decrease in disulfide bond levels (Fig. 4**d**) may be attributed to the suppression of sulfhydryl conversion. The cleavage of disulfide bonds between proteins leads to a decrease in disulfide bond content, while simultaneously causing an increase in the thiol group content. This reflects the disruption of structural features, such as cross-linking and association between MPs chains, resulting in alterations to the spatial structure of the MPs.

It is generally accepted that the unfolding of protein structures, through the exposure of internal groups, facilitates protein oxidation. However, in this study, it was observed that protein oxidation was effectively inhibited. Al-Dalali et al. reported that the presence of numerous oxidative catalysts, such as iron, myoglobin and lipids in meat, led to interactions between these components during frozen storage, making the meat particularly susceptible to oxidative processes [33]. However, as a “white meat,” chicken contains significantly lower levels of iron, myoglobin and lipids compared to other types of meat, which may explain its relatively lower degree of oxidation. Furthermore, Liu et al. found that ultrasound induceds ice crystal vibrations and cavitation bubbles, which reduced the exposure of carbonyl and hydrophobic groups, thereby minimizing the generation of radicals. Ultrasound treatment can also promote the migration of carbonyl reactive thiol groups from within the protein structure to the surface, thereby helping protect MPs [35].

Surface hydrophobicity reflects the distribution of amino acid residues on the protein surface. Bromophenol blue (BPB) forms strong hydrophobic interactions with proteins, and the strength of BPB binding reflects the degree of surface hydrophobicity [36]. The surface hydrophobicity of MPs is closely related to their oxidation and structural changes. When protein oxidation occurs, conformational changes expose buried hydrophobic groups within the protein molecule, leading to an increase in surface hydrophobicity. Therefore, surface hydrophobic interactions play a crucial role in maintaining the conformation and functional properties of MPs [37]. As shown in Fig. 4**c**, although no significant differences were observed between the groups, AMP and ultrasound treatments were found to enhance the surface hydrophobicity of CWB, indicating that both AMP and ultrasound treatments promoted the unfolding of MPs and the exposure of nonpolar amino acids on their surface. Zhu et al. found that when proteins were exposed to an aqueous environment in a salt solution at certain concentrations, hydrophilic groups formed hydrogen bonds and were exposed on the surface [37]. In contrast, hydrophobic groups tended to aggregate, reducing their contact area with water [37]. In salt solutions, both aggregation and unfolding features can occur, which also explains the increase in hydrophobic interactions observed in the saline treatment group.

### 3.5 Microscopic structure of muscle fibers

**Fig. 5:**
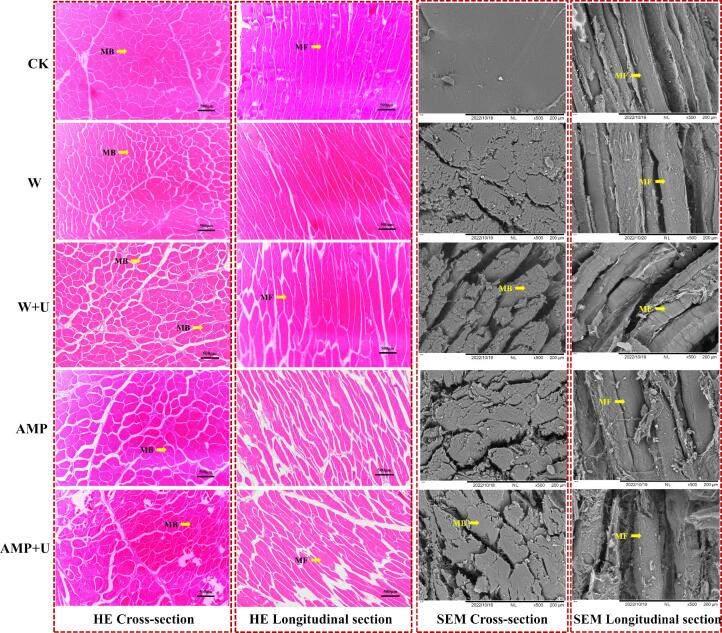
Effects of different treatments on the pathology and microstructure of chicken breast. The scale bar for histology is 500 μm. The scale bar for scanning electron microscopy (SEM) is 200 μm. MB is muscle bundle and MF is muscle fiber.

As shown in Fig. 5**,** in the control group (CK), the cross-sectional view of the tissue exhibited tightly packed muscle fibers, minimal inter-bundle gaps, and a relatively intact structure with well-preserved sarcolemma. Similarly, the longitudinal section of the CK group was characterized by a smooth, dense, and structurally intact appearance, with no evidence of fiber rupture. Following tenderization treatments, the tissue structures underwent varying degrees of alteration, with the most pronounced changes observed in the A + U group. The muscle bundles of chicken breast tissue subjected to AMP and ultrasound treatments exhibited significant irregular swelling, increased spacing, and the formation of voids in the cross-sectional view. It has been suggested that myofibril swelling represented another form of ultrastructural alteration, which may result from the absorption of water into the intramyofibrillar spaces [18].

In the longitudinal sections, muscle fibers exhibited roughened surfaces, loosened gaps, shortened lengths, and even breakages. Notably, ultrasound treatment exerted a more pronounced effect on the longitudinal muscle fibers compared to AMP, as evidenced by the coarse appearance observed in SEM images (Fig. 5). This phenomenon may be attributed to an additional significant damage induced by ultrasound, namely, the weakening and fragmentation of MPs, particularly along the Z-lines, where broken and twisted muscle fibers were observed. These effects may be driven by the Tearing forces and shock waves generated by ultrasound-induced cavitation. Furthermore, the increased brittleness of lignified muscle bundles contributes to the exposure of myofibrils, which is another critical feature observed in the analysis. Notably, the observed microstructural changes in the muscle may facilitate the release of intracellular contents and the penetration of AMP, which could enhance proteolytic enzyme activity within the tissue, thereby triggering enzymatic hydrolysis of myofibrillar structures. The separation of muscle fibers from one another and the longitudinal fragmentation of muscle fibers, as revealed through microstructural analysis, are direct contributors to meat tenderization, resulting in improved tenderness and overall eating quality. These microstructural alterations were further corroborated by the changes in the MFI (Fig. 2).

## 4 Conclusions

The combined treatment of ultrasound and AMP was found to be particularly effective in improving the tissue structure and tenderness of woody breast chicken. These improvements are primarily attributed to the synergistic effects of ultrasound-induced cavitation, which disrupts muscle fiber structure, and AMP, which activates endogenous enzymes. The discovery of AMP's role in enhancing enzymatic activity provides valuable insight into its potential as a novel additive for meat quality improvement. Similarly, ultrasound emerges as a versatile technology capable of inducing structural modifications in muscle tissues. These findings not only offer theoretical support for improving the quality of woody breast meat but also highlight promising applications of AMP and ultrasound in developing innovative processing technologies. Future studies should focus on optimizing the parameters of these treatments and exploring their efficacy in other meat products, paving the way for broader industrial applications and advancements in meat processing technology.

## CRediT authorship contribution statement

**Xiang Yu:** Writing – review & editing, Writing – original draft, Visualization, Validation, Supervision, Resources, Project administration, Methodology, Investigation, Funding acquisition, Formal analysis, Data curation, Conceptualization. **Yanli Feng:** Writing – review & editing, Writing – original draft, Software, Investigation, Funding acquisition, Formal analysis, Data curation, Conceptualization. **Wenhan Ma:** Investigation. **Xue Xiao:** Investigation. **Jun Liu:** Software, Resources, Formal analysis. **Weiwei Dong:** Software, Resources. **Yuanliang Hu:** Supervision, Resources. **Huan Liu:** Writing – review & editing, Writing – original draft, Software, Resources, Project administration, Funding acquisition.

## Funding

The Project of Natural Science Foundation of Hubei Province (Joint Fund, Project No. 2023AFD025) and Centralized Local Science and Technology Development Funds (Laboratory Major Scientific and Technological Achievement Transformation) Project (2024BSB020).

## Declaration of Competing Interest

The authors declare that they have no known competing financial interests or personal relationships that could have appeared to influence the work reported in this paper.
